# Utility of Psychometric and Dynamic Assessments for Identifying Cognitive Characteristics of Twice-Exceptional Students

**DOI:** 10.3389/fpsyg.2021.747872

**Published:** 2021-12-16

**Authors:** Anies Al-Hroub

**Affiliations:** Department of Education, American University of Beirut, Beirut, Lebanon

**Keywords:** twice exceptional, gifted and talented students, psychometric ability tests, dynamic assessment and testing, learning disabilities, mathematically gifted, cognitive abilities, perceptual skills

## Abstract

The purpose of this research was to examine the utility of psychometric and dynamic assessment for the identification of a twice-exceptional (2E) group of students who showed both mathematical high abilities and specific learning disabilities. Of a population of 800 students, 30 (14 boys and 16 girls) ages 10 to 12 years were selected and identified as twice-exceptional at three public elementary schools in Amman, the capital of Jordan. A combination of three psychometric tests and one dynamic math assessment tool was used to recognize the cognitive and perceptual characteristics strengths and difficulties among students. Both psychometric and dynamic assessment models were found important and complementary to one another for the identification of cognitive and perceptual characteristics of twice-exceptional children. The findings were reported and discussed.

## Introduction

Over the last thirty years, increasing attention has been given to the demanding question of “twice-exceptional” – 2E and mainly those who are highly able and show specific learning disabilities – LDs (Al-Hroub, 2014; Montgomery, 2015; Al-Hroub, 2020). According to Baum et al. (2017), Baum (2004), those 2E/gifted with LDs fall into at least three subgroups: (1) gifted with hidden LDs, (2) students with LDs with hidden giftedness, and (3) students with both hidden giftedness and LDs. In the last decade, Al-Hroub’s (2010a), Al-Hroub (2013) research study showed that there are two additional subgroups of 2E: (1) learners whose giftedness and LDs are both known to their teachers and parents and (2) students who are misdiagnosed with other disorders. According to Al-Hroub (2013), the latter subgroup was to be found the most at-risk because most of them are found to receive inappropriate educational services. Despite this body of research, recent studies revealed that teachers in schools are relatively poorly equipped to identify 2Es (Al-Hroub, 2007; Al-Hroub and Krayem, 2018; El Khoury and Al-Hroub, 2018).

Some theorists have argued about the lack of a universally accepted definition of mathematical talent (Al-Hroub, 1999; El Khoury and Al-Hroub, 2018). Sowell et al. (1990) argued that mathematically gifted learners are those who are precociously very able in solving mathematical problems that are typically not accomplished by students of their chronological age. Such gifted students engage in qualitatively different mathematical thinking processes than those of their classmates. Both Krutetskii (1976) and Benbow and Minor (1986) emphasized the fundamental meaning of mathematical reasoning as against the skillful use of algorithms, in differentiating mathematically gifted from non-gifted learners. Krutetskii (1976) identified two fundamental characteristic abilities present in mathematically gifted learners: (1) high levels of commitment to tasks/work and (2) high levels of originality and flexibility in solving problems. Similarly, Renzulli’s (1977) three-ring conception of giftedness identified task commitment and creativity as two fundamental elements of the cluster traits. Successively, Ball (1993) replicated Renzulli’s conception to characterize mathematically gifted learners “as those who have: (1) above-average ability; (2) creativity in math, which is the ability to respond with flexibility and creativity to a mathematical problem; and (3) task commitment in their pursuit of a solution to a mathematical problem” (Al-Hroub, 2011, p. 25).

The issue of educational and psychological assessment is essential because it determines the methods, tools, and teaching practices that will be used with learners. Special education teachers and educational psychologists who develop individual educational plans try to use the psychological assessment findings to inform the next steps of the child’s learning. Conventionally, several researchers (e.g., Al-Hroub, 2012; Kaufman et al., 2016; Al-Hroub and Whitebread, 2019) have been concerned with psychometric scales, such as cognitive, perceptual skills, and achievement tests. These scales have suffered from biases and rigid administration procedures, such as using timed tests and testing under stringent conditions (Al-Hroub, 2011). Psychometric tests have received further criticism for being unfair to vulnerable and marginalized and those with exceptionalities and 2E. Further criticism could be related to the use of general intelligence and achievement scales that lead to misdiagnosis and inaccurate labeling (Lauchlan, 2001; Lauchlan and Elliott, 2001; Shehab and Al-Hroub, 2019).

Several researchers (e.g., Sternberg and Grigorenko, 2002; Swanson and Howard, 2005; Moore-Brown et al., 2006; Haywood and Lidz, 2007; Al-Hroub and Whitebread, 2008; Al-Hroub, 2009) have suggested using the concept of dynamic assessment (DA) to assessing the potential of learners with LDs and those with high intellectual abilities. This movement toward DA was partly initiated by the Russian psychologist Vygotsky on the Zone of Proximal/Potential Development (Vygotsky, 2012). The approach is that with expert guidance (mediation) and specific delivery (scaffolding), children will be exposed to a rich environment for development in exploring new tasks, knowledge, and ideas. Rutland and Campbell (1995) argued that using DA methods is essential for identifying the untapped potential of learners with LDs since general IQ tests may underestimate the high potential of those learners. This is mainly because DA methods focus on *learning processes,* whereas static psychometric assessments are more interested in *learned products* (Haywood and Lidz, 2007).

Vygotsky’s developmental conceptions of “plus- and minus-giftedness” have led to the Dynamic Theory of Giftedness (Vygotsky, 1983). This conception uses the dynamic model that either giftedness or deficits are possible products when a learner is faced with development challenges. Therefore, the DA approach may provide a means for assessing deficits as well as high abilities. Bolig and Day (1993) explained that DA is a useful method in identifying gifted students because it can detect the individual differences in learning, provides evidence that helps determine the methods to teach exceptional learners, and tailor teaching to the learner’s preferred learning styles and distinctive personality.

In contrast, Murphy (2011), Sternberg and Grigorenko (2002), and Beckmann (2006, 2014) presented some problematic issues with the use of DA: (1) it preserves the misguided perception that DA is useful only for sub-performing populations, such as 2E learners; (2) can only be utilized for underachieving populations which have not been allowed to explore their full potential; (3) the time taken to administer clinical versions of DA are not used within school settings; (4) the proof of higher validity of DA, compared with psychometric assessment, is inadequate; and (5) the need to extensively and comprehensively train moderators and teachers within a DA set-up is cost and time-demanding.

Several specific challenges are related to assessing learning disabilities for Jordanian students in Arabic. First, Arabic is often considered a diglossic language. In teaching Arabic literacy, there is a marked differentiation between two related varieties: (1) *Fusha –* classical Arabic or modern standard Arabic (used for “high” functions) and (2) *Ammiya* – colloquial Arabic or Arabic dialects (used for “low” functions). However, Arabic diglossia seems to be a major cause of low learning achievement and hinders children’s Arabic-reading acquisition, especially in the early years (Ayari, 1996; Saiegh-Haddad and Henkin-Roitfarb, 2014). Abu-Rabia (2001) stressed that when first-grade students are introduced to literary Arabic, invariably, they find it almost a new language for reading, writing, and even speaking. Although several researchers (e.g., Ayari, 1996; Holes, 2004) claimed that children in Arab countries are not exposed to *Fusha* until they enter formal schooling, children in Jordan do have some *Fusha* exposure before entering the school through social media, television programs, and literacy events. Yet, this exposure might be relatively inadequate, depending on the child’s context (Dakwar, 2005). Second, Arabic is known as a highly homographic language (i.e., words look very similar but carry dissimilar meanings; Abu-Rabia, 2000). Third, Arabic letters require more visual attention to their discrimination than do English letters. For example, some letters have three different forms for the initial, middle, and end position in the word (Abu-Rabia, 2000; Yassin et al., 2020).

The purpose of this research study was to examine the utility of psychometric and dynamic assessment (DA) for identifying cognitive and perceptual characteristics of 2E children exhibiting mathematical giftedness and specific learning disabilities (MG/LDs) in Jordan. The specific questions guiding this study were as follow: (1) What are the cognitive characteristic patterns that MG/LD students tend to exhibit on the Wechsler Intelligence Scale for Children (WISC-III-Jordan)?, (2) to what extent does use of DA address the high mathematical abilities in learners experiencing specific learning disabilities?, (3) what are the specific visual and auditory perceptual skills of these MG/LD students?, and (4) what patterns of specific learning disabilities are displayed by MG/LDs students?

## Method and Instruments

### Sample

As multiple case studies, a team of two educational psychologists identified 30 Jordanian students (16 girls, 14 boys), aged 10 years to 11 years and 11 months, at three large public elementary schools in Amman, and the capital of Jordan. The children were chosen from fifth and sixth-grade levels to avoid earlier years when it became difficult to differentiate between learning disabilities and learning problems since students are still exposed to basic literacy skills. Students were nominated from a population of 800 fifth and sixth graders in the three schools (99 students in school 1,205 students in school 2, and 496 students in school 3). In the process of identification, 52 students were nominated by their regular classroom teachers then evaluated by two educational psychologists. Of 52 students, 22 showed learning disabilities but with average intellectual ability. Thus, we referred to this as the “Average IQ/LD” group.

### Procedures and Assessment Identification Approaches

Several assessment scales and subscales were administered, by which some were used to identify students’ mathematical giftedness (MG), whereas others were used to identify their specific learning disabilities (LD). The assessments that were used to identify the students were carried out by two certified educational psychologists, who used differentiated assessment for the identifications of student’s potential and deficits. They used IQ tests and DA for measuring high intellectual and mathematical abilities, whereas perceptual skills and literacy tests measure deficits in reading, writing, or spelling. Both psychologists are licensed to administer psychometric tests. The first psychologist administered the WISC-III-Jordan and dynamic math tests, whereas the second psychologist administered the perceptual skills tests and the diagnostic scale of Arabic Language Basic Skills. Most of the assessments were carried out in the counselors’ or learning resource rooms. The author used DA for the identification of high mathematical potential for the following reasons: (1) All nominated students (*n* = 52) were largely recognized by their Arabic and math teachers as having LDs rather than high mathematical potential, (2) a combination of psychometric (e.g., WISC-III-Jordan) and DA is essential for identifying the tapped and untapped learning potential of those nominated students, and (3) it was essential to avoid over-testing the nominated students in their areas of weaknesses, given that two psychometric tests were used to identify their LDs.

The study has adhered to the American Psychology Association’s ethical standards. Permissions were obtained from IRB and the Ministry of Education to conduct the study in the selected schools.

#### The Wechsler Intelligence Scale for Children

The researcher used the third version because it is the last version that was adapted and used in Jordan. As with all other WISC versions, the WISC-III-Jordan (1996) is an individually administered clinical instrument. It was used to assess the intellectual functioning of the children ages range between 6 years and 16 years 11 months, which fits the age group of the study participants. The scale consists of 13 subtests (10 standards and 3 supplementary) and three scales (verbal, performance, and full-IQ). To qualify for intellectually gifted status, examinees generally have to score at least 130 on the WISC (Montgomery, 2015). However, several psychologists suggested dropping the cutoff score by 10 points for 2E learners due to their specific difficulties (Silverman, 1989; Al-Hroub, 2013). In the present study, we will consider 120 as the cutoff score to qualify for the intellectual giftedness status.

The reliability was examined by the test–retest method for all of the subscales and showed high-reliability coefficients of 0.95, 0.94, and 0.96, respectively, across all ages groups. Reliability was also measured by inter-rater/scorer agreement. Verbal, Performance, and Full-Scales IQs have average reliability coefficients of 0.94, 0.88, and 0.95, respectively, across all ages (Wechsler, 1996).

This scale was administered to answer the question, “What are the cognitive characteristic patterns that MG/LD students tend to exhibit on the WISC-III-Jordan?”

#### Dynamic Math Assessment

A test-intervention-test method was employed to determine students’ high potential in math. Two equivalent pre- and post-tests were developed by the researchers. The tests were derived and developed from items taken from the Diagnostic Scale of Mathematical Basic Skills (Waqfi, 1997). This diagnostic scale is used in Jordanian schools to identify the high mathematical potential of elementary and middle school students. The DA tests covered seven mathematical areas (calculation operations, decimals, rounding up, geometry, algebra, and written problem-solving). Before administrating the tests, pilot test sessions were administered with 8 mathematically gifted students (4 boys/4 girls; 4 fifth/4 sixth graders). Those students were selected by their schools based on their high cumulative math performance. The pilot test sessions were crucial to allow revising and modifying the questions, ensuring the difficulty level, and ensuring the time required for responding to the questions (average 45 min). Yet, the DA tests administration was not restricted by time to allow identifying high mathematical potential in students who usually take their time in responding to questions.

The tests administration consisted of three phases. In phase one, the researcher administered the developed pre-test. In phase two, the researcher delivered particular teaching related to the nature of the questions and math areas in the pre-test. Three teaching sessions were delivered, 45 min each. In phase three, the post-teaching stage, another developed test was administered to assess the same math areas. The psychologist/researcher who conducted the assessment was blind to the study. They read all questions to students. This DA method was administered to answer the question, “To what extent does the use of DA address the high mathematical abilities in learners experiencing specific learning disabilities?”

#### The Group of Perceptual Skills Tests

The tests provide “a profile of the strengths and weaknesses that are often associated with specific learning disabilities of children aged from 6 years 7 months to 16 years 6 months.” (Waqfi and Kilani, 1998). The Perceptual Tests take around 45 min overall to administer, which is within the attention span of most children in this age group. The Perceptual Skills Tests battery includes seven diagnostic subtests covering the range of skills that are known to be affected in dyslexia and the profile of disabilities that can be used to interpret the causes of attainment difficulties; these subtests are as follows: (1) Auditory Discrimination Test, (2) Auditory Analysis Skills Test, (3) Word Span Test, (4) Digit Span Test, (5) Visual-Motor Sequence Test, (6) Visual-Motor Integration, and (7) Visual Analysis Skills Test (Waqfi and Kilani, 1998). However, the seven perceptual subtests were categorized into six major perceptual areas (Waqfi and Kilani, 1998), as follows: (1) *Auditory Perceptual Skills*, which consists of four subtests: (a) Auditory Discrimination, (b) Auditory Analysis Skills, (c) Auditory Word Span, and (d) Auditory Digit Span; (2) *Auditory Discrimination and Analysis Skills*, which consists of two subtests: (a) Auditory Discrimination and (b) Auditory Analysis; (3) *Auditory Short-Term Memory*, which consists of two subtests: (a) Auditory Word Span and (b) Auditory Digit Span; (4) *Visual Perceptual Skills*, which consists of three subtests: (a) Coding, (b) Visual-Motor Integration, and (c) Visual Analysis Skills; (5) *Visual Integration and Analysis Skills*, which consists of two subtests: (a) Visual Motor-Integration and (b) Visual Analysis Skills; and (6) *Visual Short-Term Memory*, which includes of the Coding subtest. Construct validity was assessed by administering the seven subtests to a group of 270 children previously diagnosed as dyslexic. The authors presented evidence of validity based on the internal structure between subtests across all age groups. All subtests were found to significantly correlate with one another, as would be expected considering that they all presumably measure “perceptual skills.” Further evidence of internal structure is presented through correlation coefficient validity of subtests across all age groups. The seven perceptual skills subtests have average validity coefficients (*r*) of 0.51, 0.73, 0.96, 0.92, 0.67, 0.95, and 0.96, respectively, across all ages. This result agrees with the performance development along with all ages’ development. A study with 270 children has shown that the inter-item reliability, using Cronbach’s alpha coefficients (*r*), is excellent (above 0.90) for most of the subtests and satisfactory (0.73 and 0.68) for Word and Digit span. The study has shown that the correlations are 0.92, 0.93, 0.73, 0.68, 0.96, 0.90, and 0.95, respectively, across all ages. Further, split-half reliability coefficient alphas were calculated using the Spearman-Brown formula. Stability coefficients for the subtests ranged from 0.71 and 0.94 (Al-Hroub, 2013, p. 57).

This Group of Perceptual Skills tests was used to answer the third research question, “What are the specific visual and auditory perceptual skills of these MG/LD students?”

#### The Diagnostic Scale of Arabic Language Basic Skills

This is a diagnostic scale that covers a comprehensive range of topics in Arabic Language skills. The scale is designed to examine the basic Arabic literacy skills of children between 6 and 15 years old (Waqfi, 1997). The researchers used seven subtests to answer questions related to the research study, namely, “(1) Vocabulary Recognition, (2) Reading Different Vocabulary, (3) Reading Similar Vocabulary, (4) Reading Comprehension Passages, (5) Listening to Comprehension Vocabularies, (6) Listening to Comprehension Passages, and (7) Spelling and Dictation. These seven subtests were categorized into one of three learning aspects: (1) *Reading Ability*. This contains four subtests: Vocabulary Recognition, Reading Different Vocabulary, Reading Similar Vocabulary, and Reading Comprehension Passages; (2) *Listening Ability*. This examines the extent to which the student can comprehend information to which he/she has listened. It contains two subtests: Listening to Different Vocabulary and Listening Comprehension Passages; and (3) *Spelling and Dictation*. This area is represented by the graded Spelling and Dictation subtest (Waqfi, 1997). The Scale has been correlated significantly with a sample of students’ achievement in the Arabic language and showed high inter-correlations, particularly in Reading Vocabularies, Passage Comprehension, Listening Comprehension Vocabularies, and Spelling, which provides evidence for the construct-related validity of the test. The predictive validity of the same edition has been thoroughly investigated. The Scale’s manual reports two main types of reliability measures for the test: stability (parallel forms), reliability, and internal consistency. The correlation coefficient between the parallel forms A and B on the number of children reading incorrect words (on all of the vocabulary scale series) suggests high stability between 0.65 and 0.89 at all grade levels. Also, the results of the correlation coefficient between the two forms on the number of students’ reading incorrect Different Vocabularies vary between 0.38 and 0.72, whereas it is between 0.34 and 0.74 for Reading Similar Vocabularies (Al-Hroub, 2013, p. 58). This diagnostic scale was administered to answer the fourth research question, “What patterns of specific learning disabilities are displayed by MG/LDs students?”

## Results

Table 1 shows scatter/range indices for the two research groups (MG/LDs and the Average-LD). The findings showed that despite the large VIQ-PIQ discrepancy (*M* = 12.73) for the MG/LDs sample, it is not significantly higher than the Average-LD group. The table also shows no significant difference between the Verbal Comprehension-Perceptual Organization. With regard to the scaled-score range on the Full Scale, significant differences level [*t*(50) = 2.09, *p* < 0.05] were identified between the 2E and none-2E groups (MGLD versus Average-IQ/LD). Medium Cohen’s effect size (*d* = 0.59) and stet correlation (*r* = 0.28) were shown, which indicates that the differences have medium practical implications.

**Table 1:** WISC-III-Jordan scatter indices within the verbal and performance scales for the MG/LDs and Average-IQ/LD groups.

WISC-III-Jordan Scatter Indices	MG/LDs (*n* = 30)		Average-IQ/LD (*n* = 22)		Independent sample *t* tests (*df* = 50)	Size Effect	
	Mean Difference	*SD*	Mean Difference	*SD*		*Cohen’s* d (2)	*r*
VIQ-PIQ discrepancy	12.73	11.04	7.95	8.06	1.72	0.49	0.24
VC-PO discrepancy	8.63	10.90	5.91	8.70	0.967	0.27	0.14
Verbal Scaled Score Ranges	4.40	1.73	4.50	1.90	−0.20	−0.06	0.03
Performance Scaled Score Ranges	5.57	2.27	5.45	1.82	0.19	0.054	0.027
Full IQ Scale	7.70	1.84	6.68	1.59	2.09^*^	0.59	0.28

d = 0.2 (‘small’ effect size), d = 0.5 (‘medium’ effect size) and d = 0.8 (‘large’ effect size;Cohen, 1988). ^*^Significant at level p < 0.05; ^**^Significant at level p < 0.01.

Table 2 reports descriptive statistics of the dynamic pre- and post-tests for the MGLDs/2E sample. The significant difference were found between the DA pre- and post-tests [*t*(29) = 25.24, *p* < 0.01]. A large Cohen’s effect size (*d* = 9.22) and stet correlation (*r* = 0.98) were identified, which indicates that the difference has high practical implications.

**Table 2:** Mathematical learning progress for the MGLDs/2E students.

Dynamic Math Tests^1^	MG/LDs Sample (*n* = 30)					Effect Size	
	Min	Max	Mean	*SD*	Paired *t*-test (df = 29)	*Cohen’s* d	*r*
Pre-Test	8.00	14.00	10.55	1.49			
Post-Test	15.0	20.0	17.63	1.30			
Mathematical Learning Progress	4.50	10.50	7.08	1.54	25.24^**^	9.22	0.98

1 *The tests were out of 20 points*.

** Significant at level p < 0.01: ^*^Significant at level *p* < 0.05;

*d = 0.2 (‘small’ effect size), d = 0.5 (‘medium’ effect size) and d = 0.8 (‘large’ effect size; Cohen, 1988)*.

The findings revealed that more than 90% of the students who scored 120 or above on the IQ test showed a high inconsistency of performance (35.4%) before and after the dynamic intervention. However, no statistically significant correlations were found between DA mathematical learning progress and the WISC Arithmetic subtest. This demonstrates that psychometric subtests, such as arithmetic, did not help identify the math potential of most 2E students.

Table 3 reports the mean score in each paired factor for the MGLD/2E group. Using paired sample *t* tests, nine paired factors were compared. The findings showed significant mean differences in seven paired tests and skills, namely, (1) auditory discrimination-auditory analysis [*t*(29) = 5.67, *p* < 0.01], (2) auditory short-term memory-auditory discrimination/Analysis [*t*(29) = − 5.44, *p* < 0.01], (3) visual-motor coordination-visual analysis skills [*t*(29) = 4.70, *p* < 0.01], (4) visual S-T memory-visual integration/analysis [*t*(29) = − 2.53, *p* < 0.05], (5) auditory discrimination-visual motor integration [*t*(29) = 2.19, *p* < 0.05], (6) auditory S-T memory-visual S-T memory [*t*(29) = − 2.85, *p* < 0.01], and (7) auditory perceptual-visual perceptual skills [*t*(29) = − 3.28, *p* < 0.01]. The findings were supported by a small to medium Cohen’s effect size for all significant differences shown in paired factors (see Table 3). No significant differences were reported for auditory word span – auditory digit span and auditory analysis skills – visual analysis skills.

**Table 3:** Perceptual skills for the MGLDs/2E students.

Skills versus Skills	Paired Factors	MG/LDs (*n* = 30)			Effect Size	
		Mean Difference	*SD*	Paired Sample *t-*test (df = 29)	*Cohen*’s d	*r*
Auditory vs. Auditory Tests and/or Skills	Auditory Discrimination – Auditory Analysis Skills	11.90	11.51	5.67^**^	2.11	0.73
	Auditory Word Span – Auditory Digit Span	3.43	10.97	1.71	0.64	0.30
	Auditory Short-Term Memory – Auditory Discrimination / Analysis Skills	−8.10	8.16	−5.44^**^	−2.02	0.71
Visual vs. Visual Tests and/or Skills	Visual Motor Integration – Visual Analysis Skills	6.13	7.14	4.70^**^	1.75	0.68
	Visual Short-Term Memory – Visual Integration/Analysis Skills	−3.50	7.58	−2.53^*^	−0.94	0.43
Auditory vs. Visual Tests and/or Skills	Auditory Analysis Skills – Visual Analysis Skills	−3.57	10.28	−1.90	−0.71	0.33
	Auditory Discrimination – Visual Motor Integration	2.20	5.49	2.19^*^	0.81	0.38
	Auditory Short-Term Memory – Visual Short-Term Memory	−5.28	10.16	−2.85^**^	−1.06	0.47
	Auditory Perceptual Skills – Visual Perceptual Skills	−3.57	5.96	−3.28^**^	−1.21	0.52

* *Significant at level p < 0.05*;

** *Significant at level p < 0.01*.

*d = 0.2 (‘small’ effect size), d = 0.5 (‘medium’ effect size) and d = 0.8 (‘large’ effect size; Cohen, 1988)*.

The findings also showed that the MGLDs/2E students fall into four subgroups: (1) students with auditory processing skills difficulties (40%); (2) students with visual processing skills difficulties (6.7%); (3) students with mixed auditory and visual skills difficulties (40%); and (4) students with no apparent perceptual problems (13.3%). With regard to short-term (S-T) memory skills, the findings placed the MGLDs/2E in four subgroups: (1) 26.7% with poor visual S-T memory; (2) only 3.3% with poor visual S-T memory; (3) 63.3% with poor visual/auditory S-T memory; and (4) 6.7% with auditory S-T memory. These findings revealed that most difficulties fall under the auditory processing skills and mixed auditory and visual skills. Also, poor visual and auditory S-T memory were found among most 2E children.

Finally, the results indicated significant differences between the visual and auditory S-T memory. Also, students scored above average on auditory discrimination, low average on auditory analysis, high average on visual-motor integration, and a low average on visual analysis.

MG/LDs students showed poor literacy skills in spelling, writing, and listening. Reading Ability was the weakest area of literacy regardless of gender. Students showed severe literacy delay, between 1.2 and 2.5 grades, in all areas.

## Discussion

Three critical issues emerged from the data analysis of the research. These issues are the utility of psychometric testing for the identification of MG/LDs, the utility of DA for identification of MG/LDs, and psychometric versus DA.

### Utility of Psychometric Assessment for Identification of Twice-Exceptional

The analysis of the cognitive and perceptual skills characteristics of 2E learners (MG/LDs), in the present study, suggests some general and specific implications. As a general result, 2E learners demonstrated high visual and verbal abilities. These findings suggest that most 2E students exhibiting high mathematical abilities and learning disabilities have harmonic mathematical abilities, as stated by Krutetskii (1976).

With regard to the Verbal-Performance IQ discrepancy, the findings support the argument that such traditional formula may not be the best indicator for LDs (Kaufman et al., 2016). But, it could be used as a good indicator of the co-existence of LDs and mathematical giftedness in the same person (Al-Hroub, 2010b, 2014). However, Bray et al. (1998, p. 212) noted that “although a discrepancy of 11 points between Verbal and Performance IQ scores is significant at the 0.05 level for all ages, it occurs in 40.5% of the standardization sample on the WISC-III.”

In regard to perceptual skills, 80% of 2E learners suffered from either auditory skills problems or a combination of visual and auditory perceptual problems. These findings correspond with a classic study done by Boder (1973), which reported that 85% of students with dyslexia showed either auditory processing skills difficulties or mixed auditory and visual processing difficulties. However, it is important to note that few studies, if any, were done on the perceptual skills of 2E learners, and Broder’s study was done on a group of only LD students.

The 2E (MG/LDs) learners showed significantly higher visual S-T memory (average skills) than auditory S-T memory (below-average skills). While little was done in the field of 2E on visual and auditory S-Term memory, a review of several case studies by Linda Silverman (2002) showed that 2E learners tend to show stronger visual memory and visual–spatial abilities. However, recent research on dyslexia indicated that a combination of audiovisual aids is crucial for dyslexic readers (see Tejero et al., 2020).

The findings revealed that students exhibited a range of LDs, which were more evident in reading and spelling. These findings were consistent with a study done in Jordan by Abu-Hamour and Al-Hmouz (2016). The problem of Arabic diglossia seemed to influence the acquisition of reading and spelling skills for Arab and Jordanian students. Moreover, the lack of diacritics (vowelized reading texts) makes Arabic reading less transparent and exacerbates difficulties in learning to read. In addition, the mixture of language patterns (e.g., *Fusha* and *Ammiya* code-switching) in educational settings is a cause of pedagogical problems, sometimes leading to inadequate reading and language competencies (Saiegh-Haddad and Henkin-Roitfarb, 2014; Sayahi, 2015).

Despite the discussion above, it would be hard to conclude that the students in the present study are not students with LDs for two main reasons. First, the findings of the two Perceptual Skills Tests and Arabic Literacy Language Skills tests were consistent in revealing students’ disabilities in reading and literacy. Second, students were exposed to Arabic literacy, *Fusha,* and vowelized reading texts for at least 5 years during their elementary education.

### The Utility of DA for Identification of MG/LDs

The findings of DA (see Table 2) suggest that there was substantial progress in performance between the pre- and post-test for the 2E students (MG/LDs). The pre-test was a good predictor of the change in scores, accounting for 90.4% (30 students from a total of 32) of the variance in performance between the pre- and post-tests. However, the progress score was the next and major factor in predicting the progress in performance, accounting for 35.4% (7.08 points) of the variance score between the pre-test and post-test. This suggests that the 2E students learned rapidly from the support they received within the intervention phase. The ceiling effect did not keep the students’ post-test scores close to their pre-test scores. Consequently, it could be concluded that both the pre-test and mathematical progress scores were the best predictors of the students’ mathematical giftedness.

### From Psychometric to Dynamic: A Continuum of Assessment

Using multiple sources of data was essential to strengthen the findings and conclusions. Although no single source was able to identify the MG/LDs, every single source was complementary to the others, and it helped to use all of the sources together. For example, the findings regarding math achievements in terms of the dynamic interaction between the students and their opportunity to learn added valid results to their psycho-educational assessment involving the WISC-III-Jordan and other specific learning disabilities tests.

A study done by Al-Hroub (2011) examined the efficacy of psychometric and DA on five MG/LDs students in the U.K. Al-Hroub used multidimensional assessment, which combined several psychometric and dynamic assessment methods, to identify multiple cases of 2E learners. The findings of the present research support this approach to providing a more evident and accurate diagnosis for each student so that they can receive the proper and appropriate training. The findings also demonstrated that dynamic measures are good predictors of mathematical potential. Thus, it is important to note that DA methods should not be viewed in direct opposition to static psychometric techniques. In contrast, the present research recommends that researchers benefit from the administration of static psychometric and standardized assessment to develop their skills in using concrete hints, gestures, and/or gradual prompts to use unstandardized mediation and adapt the educational tasks into a hands-on, concrete, and interactive pre-intervention test format for exceptional and 2E learners.

### Limitations of the Study

There are some limitations to this research study. First, the study sample should not be considered as representative of all 2E populations (e.g., gifted/ADHD and gifted/autism spectrum disorders). Second, the WISC-III-Jordan was the last developed version in Arabic in Jordan. However, we were not interested in examining the validity of the WISC-III rather to use it as a psychometric tool to identifying student’s intellectual abilities. Finally, it would have been ideal if DA was also implemented with a control group of 2E students. However, the identification process lasted for 7 months, so it was extremely difficult to identify a comparable group of 2e learners with similar cognitive and perceptual skills characteristics. Also, the focus of the paper focus was on the 2E sample rather than the LD sample, yet we had to show that during the identification process, we recognized 22 students with learning disabilities who do not fall under the operational definition of mathematical giftedness definition.

## Data Availability Statement

The original contributions presented in the study are included in the article/supplementary material, further inquiries can be directed to the corresponding author.

## Ethics Statement

The studies involving human participants were reviewed and approved by The University of Cambridge, Faculty of Education. Written informed consent to participate in this study was provided by the participants’ legal guardian/next of kin.

## Author Contributions

The author confirms being the sole contributor of this work and has approved it for publication.

## Conflict of Interest

The author declares that the research was conducted in the absence of any commercial or financial relationships that could be construed as a potential conflict of interest.

## Author’s Note

This research explores critical issues related to the identification of untapped potential among a group of twice-exceptional learners who remain unrecognized because of the inadequate use of identification and assessment methods. Twice-exceptional learners (e.g., gifted with learning disabilities) suffer from being neglected and unserved at schools because many teachers, educators, parents, and even specialists fail to recognize their strengths and/or weaknesses at schools. They are not identified as those who show both high abilities and disabilities. Thus, we discuss in this empirical research, done in a non-western, the complementary approach of using dynamic and psychometric assessment for the identification of this group of the marginalized and vulnerable population. It is essential that we discuss such a new model of identification based on research evidence done in the Middle East and the South Global about twice-exceptionality. This makes the topic very relevant to the special issue that focuses on marginalized and vulnerable populations, given that many of them are round in developing countries, the location of the research.

## Publisher’s Note

All claims expressed in this article are solely those of the authors and do not necessarily represent those of their affiliated organizations, or those of the publisher, the editors and the reviewers. Any product that may be evaluated in this article, or claim that may be made by its manufacturer, is not guaranteed or endorsed by the publisher.
